# Non-surgical Periodontal Treatment Restored the Gut Microbiota and Intestinal Barrier in Apolipoprotein E^−/−^ Mice With Periodontitis

**DOI:** 10.3389/fcimb.2020.00498

**Published:** 2020-09-21

**Authors:** Yuezhen Huang, Ying Liao, Binyan Luo, Lili Li, Yangheng Zhang, Fuhua Yan

**Affiliations:** Nanjing Stomatological Hospital, Medical School of Nanjing University, Nanjing, China

**Keywords:** periodontitis, gut microbiota, non-surgical periodontal treatment, hyperlipidemia, intestinal barrier

## Abstract

Periodontitis has been associated with a variety of systematic diseases via affecting gut microbiota. However, the influence of periodontal treatment on intestinal microbiota is not known. Hyperlipidemia can significantly alter gut microbiota structure. It is proposed that the presence of hyperlipidemia can influence the impact of periodontitis on microbiota. This study was conducted to explore the influence of periodontitis and periodontal treatment on the gut microbiota on the basis of hyperlipidemia. Apolipoprotein E^−/−^(ApoE^−/−^) mice were ligatured to induced periodontitis and non-surgical periodontal treatment was performed for half of them after 4 weeks of ligation. Microbiota communities in the feces collected at 4, 5, 8 weeks after ligation were investigated using next-generation sequencing of 16S rDNA. Bone loss at periodontitis sites were analyzed using micro-computed tomography (Micro-CT). Morphology and mucosal architecture injury of ileum tissue were observed with hematoxylin-eosin staining. The serum lipid levels were assayed. The results showed that β-diversity index in experimental periodontitis group was differed significantly from that of the control group. Significant differences were found in β-diversity between the non-surgical periodontal treatment group and the ligation group. The samples of the non-surgical periodontal treatment group and the control group were clustered together 4 weeks after periodontal treatment. Intestinal villus height and ratio of villus height to crypt depth was found decreased after ligation and restored after non-surgical periodontal treatment. Non-surgical periodontal treatment induced the colonization and prosper of butyrate-producing bacteria *Eubacterium*, which was absent/not present in the ligation group. We confirmed that periodontitis led to gut microbiota dysbiosis in mice with hyperlipidemia. Non-surgical periodontal treatment had the trend to normalize the gut microbiota and improved the intestinal mucosal barrier impaired by periodontitis in apoE^−/−^ mice.

## Introduction

Periodontal disease is a chronic inflammation of the periodontal supporting tissues resulting from the dysbiosis of the dental biofilm that causes alveolar bone loss (Page et al., 1997). Periodontitis is linked to several systemic inflammatory diseases such as obesity (Keller et al., 2015), cardiovascular diseases (Tonetti et al., 2013), diabetes (Borgnakke et al., 2013), metabolic syndrome (Kaye et al., 2016), and cancer (Ahn et al., 2012). On the other hand, The relationship between the gut microbiome and specific disease such as cancer (Scanlan et al., 2008), metabolic syndromes like obesity (Delzenne et al., 2011), diabetes (Qin et al., 2012), arteriosclerotic diseases (Koeth et al., 2013) has recently been underlined. Interestingly, these diseases are often described in association with periodontal disease (Keller et al., 2015). Subgingival biofilms in individuals with periodontal diseases present continually reservoirs of gram-negative bacteria (Li et al., 2000). It is reported that species associated with periodontitis showed evidence for mouth-to-gut transmission (Schmidt et al., 2019). Swallowing high doses of periodontal pathogenic microorganisms for a long time might induce dysbiosis of the intestinal microbiota. So far, only one clinical study reported that the abundance of phyla *Firmicutes, Proteobacteria, Verrucomicrobia*, and *Euryarchaeota* was increased in patients with periodontitis compared to the healthy control (Lourenςo et al., 2018). However, these differences were not significant (Lourenςo et al., 2018). Several studies in mice have shown that oral administration of periodontopathic bacteria *Porphyromonas gingivalis* significantly change the gut microbiota composition while induce local and systemic inflammation (Arimatsu et al., 2014; Nakajima et al., 2015; Kato et al., 2018). Thus, another possible mechanism linking inflammatory systemic diseases and periodontitis could be a disturbance of the gut microbiome.

It has been reported that there are marked changes in the gut microbiota of mice with hyperlipidemia (Hamilton et al., 2015; Liu et al., 2018) while the periodontitis alone does not significantly alter the intestinal flora (Lourenςo et al., 2018). The microbes in the gut have a profound influence on human health and disease (Cho and Blaser, 2012). However, the influence of periodontitis on gut microbiota on the basis of hyperlipidemia is not known. Mouth-to-gut microbial transmission happens more frequently in patients with chronic inflammation situations such as bowel cancer and rheumatoid arthritis (Schmidt et al., 2019). Hyperlipidemia can also enhance systemic inflammation. Therefore, we speculate that more mouth-to-gut microbial transmission happens in patients with hyperlipidemia. Moreover, when the transmission involves high proportion of periodontal pathogenic microbiota due to the patients' periodontitis, the gut microbiota could be affected to a even greater level. On the other hand, Hyperlipidemia is considered as one of the major risk factors of cardiovascular disease which is set to become the major cause of death worldwide (Satoh et al., 2009). The relation between periodontal disease and hyperlipidemia is bi-directional (Fentoglu and Bozkurt, 2008). Periodontal disease is likely to be an underlying factor for hyperlipidemia (Cutler et al., 1999; Zhou et al., 2015). On the other hand, hyperlipidemia increases the risk of periodontitis (Awartani and Atassi, 2010; Blasco-Baque et al., 2012; Zhou et al., 2015). Thus, periodontitis and hyperlipidemia are likely to occur at the same time. It makes sense to study the influence of periodontitis on gut microbiota under hyperlipidemia background.

Conventional periodontal treatment includes non-surgical approaches which enables the removal of pathogenic dental biofilms (Tsang et al., 2018). More oral microbiota transmits to the intestine under chronic inflammation situations such as bowel cancer and rheumatoid arthritis (Schmidt et al., 2019). Thus, more attention is required for the prevention and treatment of periodontal disease in patients with these diseases. It has been reported that after colonizing the intestinal flora of ordinary mice in sterile mice, the overall lipids of sterile mice increased by 60%, suggesting that the gut flora plays an important role in lipid metabolism (Bäckhed et al., 2004). Previous studies have shown that periodontal non-surgical periodontal treatment improves serum lipid levels and thus alleviates hyperlipidemia (Duan et al., 2009; Fu et al., 2016). We speculate that this is due to the recovery of gut microbiota to healthy status after non-surgical periodontal treatment.

We aimed to determine the impact of periodontitis on the gut microbiome of the mice with hyperlipidemia and evaluate the influence of the non-surgical periodontal treatment on the disturbed gut microbiome. This research reinforced the role of non-surgical periodontal treatment as an important therapy for periodontitis patients with systemic diseases.

## Materials and Methods

### Animals

Twenty-two 7-week-old male apoE^−/−^ mice (purchased from Sino-British SIPPR/BK Lab. Animal Ltd) were included in the study. All mice were housed in a specific pathogen-free facility under controlled temperatures (24 ± 1.0°C) with a 12-h light/dark cycle and were provided standard laboratory mice chow and aseptic water. All experiments were performed in accordance with the Ethics Committee of Nanjing Stomatological Hospital Medical School of Nanjing University, Nanjing, China [no. 2019NL-008(KS)]. Body weight were monitored at weekly intervals during the treatment period. The plasma total triglycerides (TG), total cholesterol (TC), low-density lipoprotein cholesterol (LDL-C), high-density lipoprotein cholesterol (HDL-C), and total cholesterol to high-density lipoprotein cholesterol (TC/HDL-C) were measured at 8 weeks from the beginning of experiment with a dry chemical analyzer (Changsha ZhongshengZhongjie Bio-Technology Co., Ltd., China). Fresh feces (0.2 g) of all mice were separately collected at 4, 5, 8 weeks after ligation. Grasping the skin of mouse's back and neck, using a 1.5-mL sterile eppendorf tubes to connect the fresh feces discharged and stored the feces at −80°C prior to processing.

At the end of the experiment, all mice were sacrificed by a sodium pentobarbital overdose. Removing the mandible, using a knife to separate the maxilla along the midpalatal suture, then cut off the right maxilla and fixed in 4% paraformaldehyde for 48 h. A 1 cm segment of distal ileum were removed and fixed in 4% paraformaldehyde for 48 h.

### Induction of Periodontitis and Non-surgical Periodontal Treatment

After 1 week of environment acclimation, the mice were randomly divided into 2 groups: experimental periodontitis group (E group, *n* = 16) and no-treatment control group (Con group, *n* = 6). In E group, silk sutures (Jinghuan Medical Appliance, Yang Zhou, China) were tied around the both sides of second maxillary molars to build ligature-induced periodontitis model (Abe and Hajishengallis, 2013). The ligatures were checked once other day.

**Figure 1:**
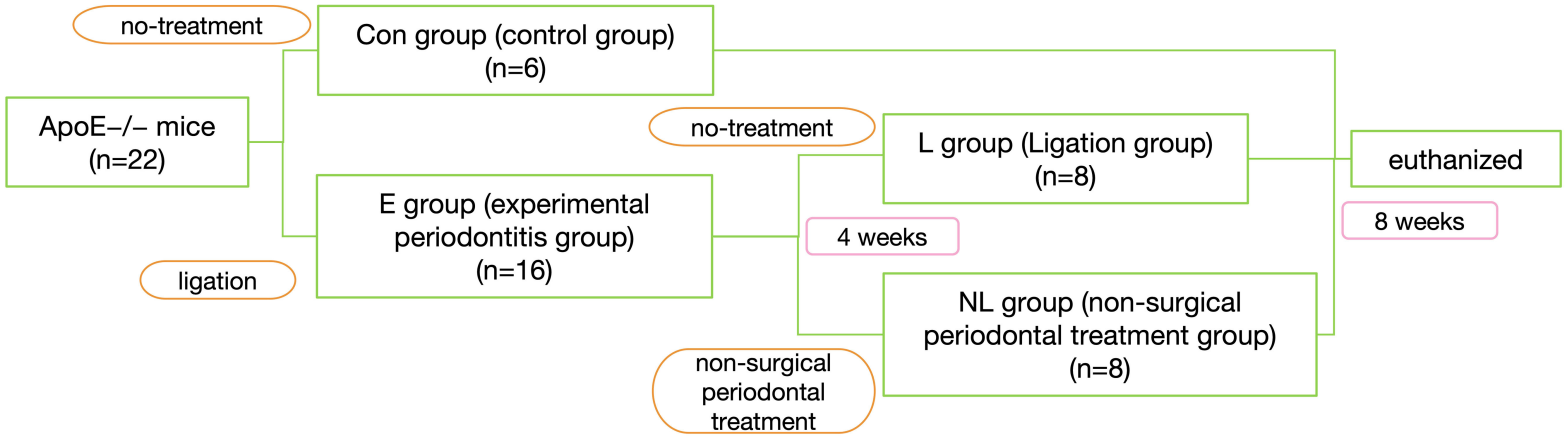
Experimental flowchart.

Four weeks after ligation, the mice of E group were randomly regrouping into 2 groups: Ligation group (L group, *n* = 8) and non-surgical periodontal treatment group (NL group, *n* = 8), In NL group, the ligatures were removed. The 2nd maxillary molars were subjected to SRP with manual 12 micro mini-five curettes (Hu-Friedy Co. Inc., Chicago, IL, USA) through 10 distal-mesial traction movements in the buccal and palate aspects. The furcation and interproximal areas were scaled with the same curettes using cervico–occlusal traction movements (de Almeida et al., 2008). All the procedures were performed by the same experienced operator. For L group, we do no treatment. 8 weeks after ligation, all mice were sacrificed by a sodium pentobarbital overdose (Figure 1).

### DNA Extraction, Amplification, and Sequencing

Total fecal DNA was extracted using the QIAamp Fast DNA Stool Minikit (Qiagen, Hilden, Germany) according to manufacturer's protocols. The V3–V4 region of the bacteria 16S ribosomal RNA genes were amplified by PCR(95°C for 3 min, followed by 30 cycles at 98°C for 20 s, 58 °C for 15 s, and 72 °C for 20 s and a final extension at 72°C for 5 min) using primers 341F 5′-CCTACGGGRSGCAGCAG-3′ and 806R 5′-GGACTACVVGGGTATCTAATC-3′. PCR reactions were performed in 30 μL mixture containing 15 μL of 2 × KAPA Library Amplification ReadyMix, 1 μL of each primer (10 μM), 50 ng of template DNA and ddH2O.

Amplicons were extracted from 2% agarose gels and purified using the AxyPrep DNA Gel Extraction Kit (Axygen Biosciences, Union City, CA, U.S.) according to the manufacturer's instructions and quantified using Qubit®2.0 (Invitrogen, U.S.). After preparation of library, these tags were sequenced on MiSeq platform (Illumina, Inc., CA, USA) for paired end reads of 250 bp, which were overlapped on their three ends for concatenation into original longer tags. DNA extraction, Library construction and sequencing were conducted at Realbio Genomics Institute (Shanghai, China).

### Micro-CT Analyses of Alveolar Bone

Micro–computed tomography analyses were performed with the method previously described with minor modifications (Papathanasiou et al., 2016). Fixed maxillae of each group were randomly selected and scanned with a Micro-CT (SkyScan 1176, Bruker micro-CT, Kontich, Belgium) at the voxel resolution of 9 μm. The 3-dimensional reconstruction (buccal view) was made to qualitatively depict the alveolar bone loss (CTvox software and DataViewer software). Mesial and distal bone loss around the maxillary second molars were quantified by measuring the distances from cement-enamel junction to the alveolar bone crest at six sites around the teeth (Image J 6.0 software).

### Morphology of Ileum Tissue

After fixation in 4% paraformaldehyde for 48 h, the ileum tissues were embedded in paraffin and cut into 5–8 μm sections. These samples were stained with hematoxylin and eosin. Images were randomly captured under the 3D HISTECH Slide Scanners (Hungary) at 160 × magnification. Five horizons of intestinal mucosa were randomly tested in each HE staining slices. Intestinal villus height (mm), intestinal crypt depth (mm) and the ratio of villus height to crypt depth (V/C) was evaluated by QUPath. Intestinal villus height is defined as the distance from intestinal villus tip to the associated intestinal crypt mouth, and intestinal crypt depth is defined as the distance from intestinal crypt mouth to the base (Liu et al., 2008). The villus height and crypt depth of three intact, well-oriented villi were measured per section (Lei et al., 2014). The intestinal mucosal damage was evaluated by using the Chiu score method (Chiu et al., 1970). Higher scores are interpreted to indicate more severe damage. Criteria of Chiu grading system consists of five subdivisions according to the changes of villus and gland of intestinal mucosa: Grade 0, normal mucosal villi. Grade 1, development of subepithelial Gruenhagen's space, usually at the apex of the villus; often with capillary congestion. Grade 2, extension of the subepithelial space with moderate lifting of epithelial layer from the lamina propria. Grade 3, massive epithelial lifting down the sides of villi. A few tips may be denuded. Grade 4, denuded villi with lamina propria and dilated capillaries exposed. Increased cellularity of lamina propria may be noted. Grade 5, digestion and disintegration of lamina proprietary; hemorrhage; and ulceration.

### Process and Analyses of Sequencing Data

Tags, trimmed of barcodes and primers, were further checked on their rest lengths and average base quality. 16S tags were restricted between 220 and 500 bp such that the average Phred score of bases was no worse than 20 (Q20) and no more than 3 ambiguous N. The copy number of tags was enumerated and redundancy of repeated tags was removed. Only the tags with frequency more than 1, which tend to be more reliable, were clustered into Operational Taxonomic Units (OTUs), each of which had a representative tag. OTUs were clustered with 97% similarity using UPARSE and chimeric sequences were identified and removed using Usearch (version 7.0). Each representative tags was assigned to a taxa by RDP Classifier against the RDP database using confidence threshold of 0.8. OTU profiling table and alpha/beta diversity analyses were also achieved by python scripts of Qiime.

Alpha diversity was assessed by the Chao 1 index. Wilcoxon signed–rank test was used for alpha diversity comparisons. To visualize the beta diversity between groups, the unweighted UniFrac distances (Lozupone et al., 2006) were calculated and plotted via principal coordinate analysis (PCoA). Adonis (PERMANOVA) testing was used to confirm significant differences in microbial community composition. Heatmap was created using R. Cluster analysis. Linear discriminant analysis (LDA) effect size (LEfSe) analysis was used to identify differential bacterial taxa representing between groups at the genus level. Thresholds for the LEfSe analysis was log LDA score >2. Kruskal–Wallis test was used to identify genera with significant differences (*p* < 0.05) among groups. Phylogenetic Investigation of Communities by Reconstruction of Unobserved States (PICRUSt) was used to predict metagenome function from the 16S rRNA data (Langille et al., 2013). Correlation analyses between the relative abundances of bacterial taxa at genus levels and alveolar bone resorption were performed by the Spearman correlation coefficient test. The Spearman correlation heat map was drawn by the corrplot package in R software. The top 30 genera with differential abundance were selected, and the Spearman correlation test was used to analyze the relationships among the dominant taxa. The Spearman correlation heat map was drawn by the corrplot package in R software.

### Ethics Statement

All the animal experiments were approved by the Animal Ethics Committee of Nanjing University and were carried out in accordance with the National Institutes of Health guide for the care and use of Laboratory animals.

### Statistical Analyses

All statistical computations were performed using GraphPad Prism 6.0 software, All the data are shown as the mean±standard deviation (SD). Differences between two groups were assessed using the unpaired two-tailed Student's *t*-test. Data sets involving more than two groups were assessed by one-way ANOVA. The differences were considered significant if *P* < 0.05.

## Results

### Non-surgical Periodontal Treatment Ameliorates the Ligature-Induced-Periodontitis in apoE^−/−^ Mice

**Figure 2:**
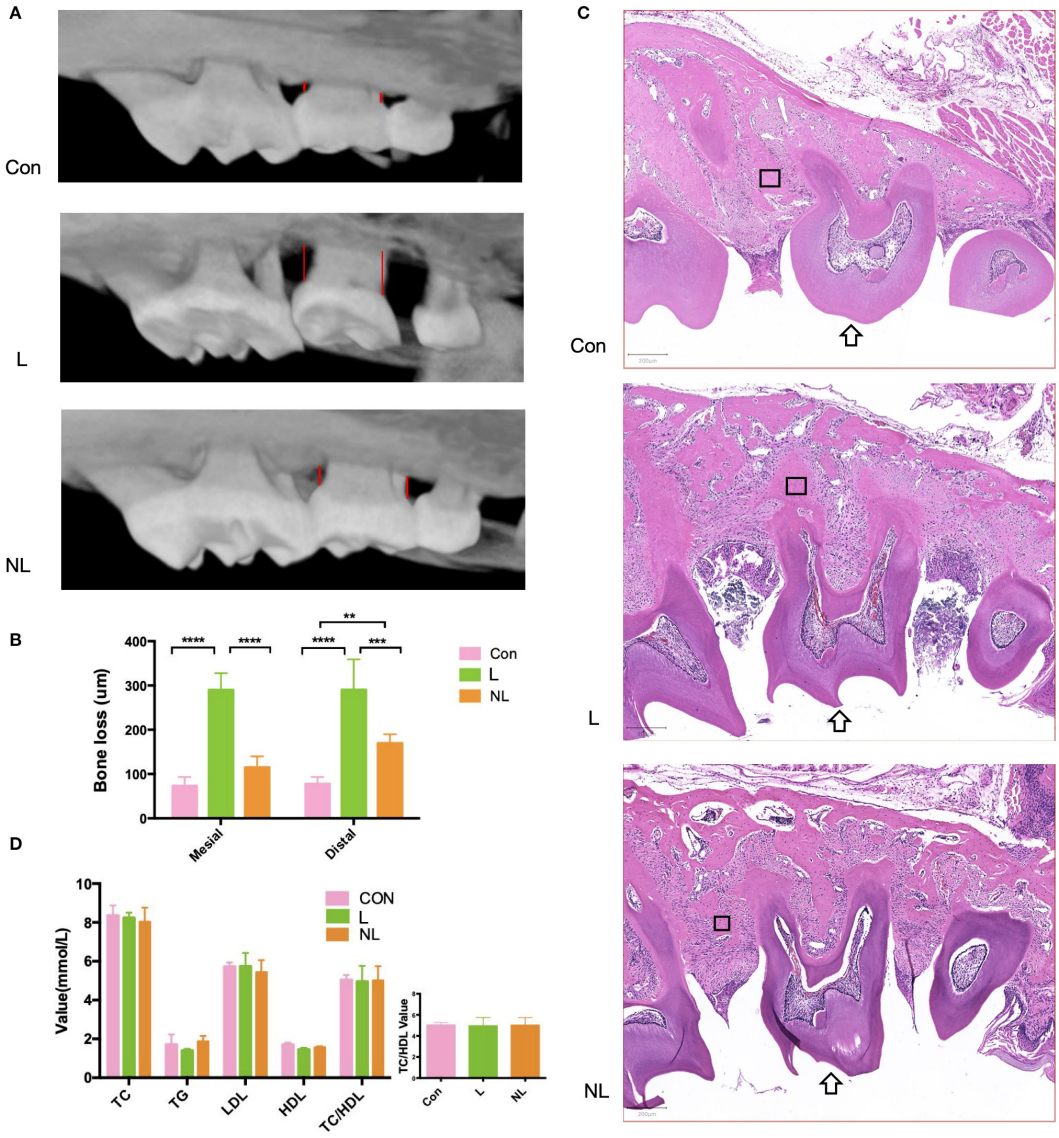
Effects of ligature placement and non-surgical periodontal treatment on periodontal tissue and serum lipid profile 8 weeks after ligation. **(A)** Micro–CT reconstruction shows alveolar bone loss at the interproximal sites (red line) of maxillary second molars (buccal view). **(B)** Mesial/distal bone loss by micro CT. Values are presented as mean ± SD (*n* = 5–8 per group). (****P < 0.0001;***P < 0.001;**P < 0.01). **(C)** Hematoxylin and eosin staining of maxillary second molar(arrow). Magnification: × 50. Scale bars, 200 μm. **(D)**TC, TG, HDL-C, LDL-C, and TC/HDL-C levels in the plasma samples from three groups of mice (*n* = 5–8 per group). Con, control group; L, ligation group; NL, non-surgical periodontal treatment group; TG, total triglycerides; TC, total cholesterol; LDL-C, low-density lipoprotein cholesterol; HDL-C, high-density lipoprotein cholesterol; TC/HDL-C, total cholesterol to high-density lipoprotein cholesterol total cholesterol. The rectangle marks the alveolar bone.

The L group showed significantly deeper periodontal pocket, elevated alveolar bone resorption, and more severe inflammation when compared to the control group (Figures 2A–C; *p* < 0.00001). After non-surgical periodontal treatment, the alveolar bone level was partly restored and inflammation was alleviated (Figures 2A–C; *p* < 0.0001). Levels of TC, TG, HDL- C, LDL-C, and TC/HDL-C did not change significantly during the whole process (Figure 2D; *p* > 0.05).

### Ligature-Induced-Periodontitis Alters the Gut Microbiota of apoE^−/−^ Mice

**Figure 3:**
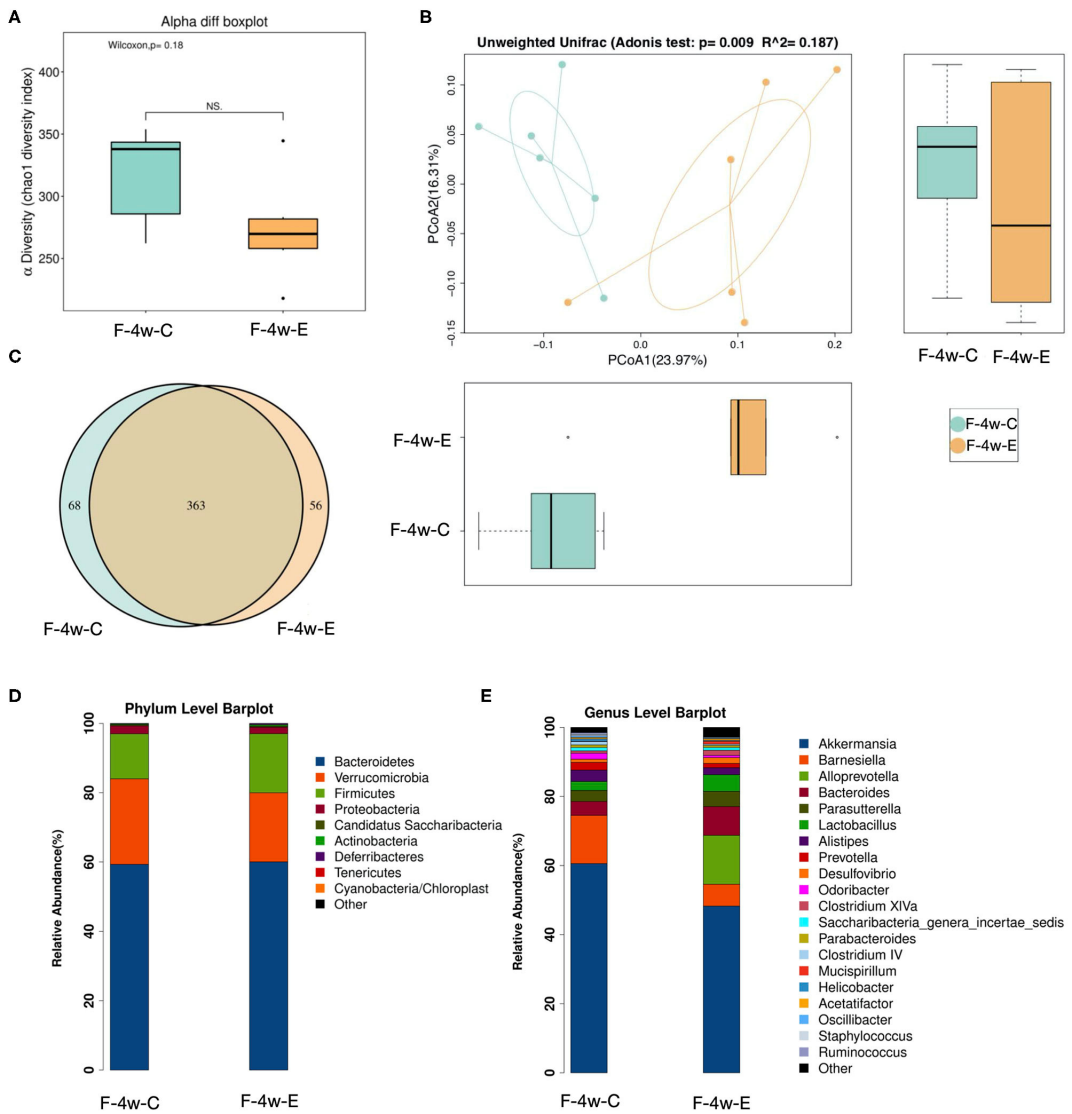
Sequencing analysis of the fecal microbiota obtained from ligatured and no treatment mice 4 weeks after ligation. **(A)** Diversity of bacterial species as indicated by Chao1 rarefaction measure. Each box plot represents the median, interquartile range, minimum, and maximum values. **(B)** PCoA of fecal microbiota from ligatured and no treatment mice. (Unweighted distance: R^2^ = 0.187, *p* = 0.009 < 0.01, Adonis test). **(C)** Venn analysis of bacterial OTUs composition between two groups (*n* = 6). **(D–E)** Proportional taxonomic assignments at the phylum level and genus level in stool samples from two groups. Only genus detected at mean relative abundance top 20 are presented. OTUs, operational taxonomic units; ANOSIM, analyses of similarities; PCOA, principal coordinates analysis; F-4w-E, experimental periodontitis group; F-4w-C, control group.

Four weeks after ligation, a lower alpha-diversity in the gut microbiome of the E group was observed, although no significant difference between groups was found (Figure 3A; *p* = 0.18 > 0.01). Beta-diversity analysis compared bacterial communities based on their compositional structures and resulted in a PCoA (distance matrix). PCoA of unweighted UniFrac distance between samples revealed that periodontitis had a profound impact on the gut microbiota composition (Unweighted distance: R∧^2^ = 0.187, *p* = 0.009 < 0.01, Adonis test; Figure 3B). Figures 3D,E showed the relative abundance of bacterial taxa at the phylum and genus level in mice from two groups. The phyla *Firmicutes* showed a tendency to increase in abundance in the experimental periodontitis group compared to the control group (Figure 3D). Greater changes of microbiota at the genus level were also observed. *Akkermansia* and *Barnesiella* showed a tendency to decrease in abundance in the experimental periodontitis group compared to the control group (Figure 3E). A Venn diagram was used to reveal the shared and unique microbiota present in the control and experimental periodontitis group. Venn diagram showed that the control group and E group had 363 bacterial taxa in common, while the control group and E group had 68 and 56 unique taxa, respectively (Figure 3C).

### Non-surgical Periodontal Treatment Modulates the Gut Microbiota of apoE^−/−^ Mice With Periodontitis

**Figure 4:**
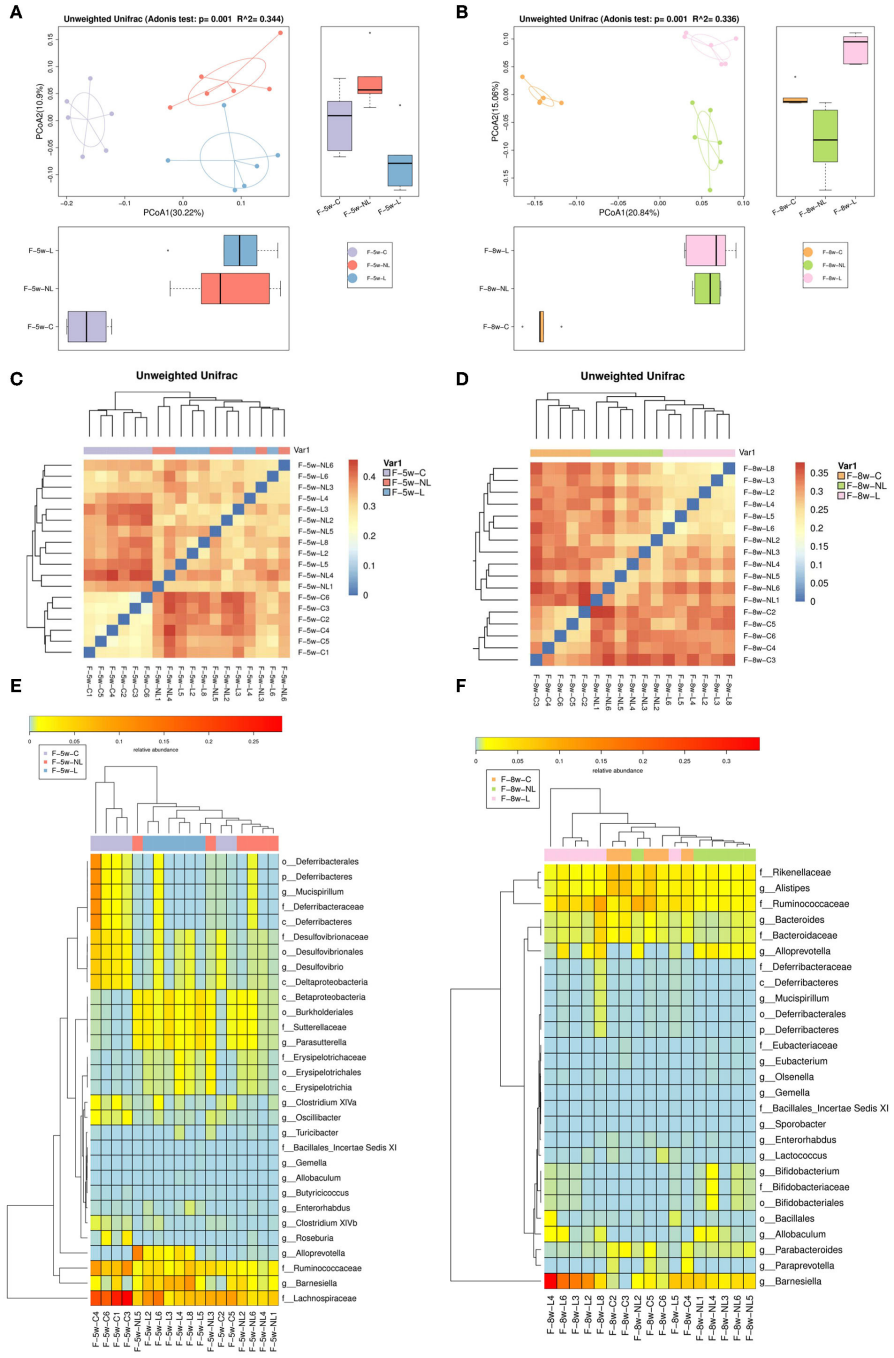
Comparison of gut microbiota among the non-surgical periodontal treatment group, the ligation group and the control group in 5 and 8 w. **(A,B)** Gut microbiota structure was shown by PCoA. (5 week: R^2^ = 0.344, *p* = 0.001; 8week: R^2^ = 0.336, *p* = 0.001, Adonis test). **(C,D)** Heatmap calculated from the unweighted UniFrac distance of the fecal samples of the three groups. **(E,F)** Heatmap cluster analysis of bacteria at all levels which have a contribution to group differences. ANOSIM, analyses of similarities; PCOA, principal coordinates analysis; F-8w-C, control group; F-8w-L, ligation group; F-8w-NL, non-surgical periodontal treatment group.

PCoA based on Unweighted Unifrac indicated that the composition of intestinal microbiome significantly altered after non-surgical periodontal treatment (5 week:R∧^2^ = 0.344, *p* = 0.001; 8 week: R∧^2^ = 0.336, *p* = 0.001, Adonis test) (Figures 4A,B). Heatmap was applied to evaluate the similarity of each sample composition. One week after the non-surgical periodontal treatment, there was no significant difference observed in the dissimilarity of bacterial structures between the non-surgical periodontal treatment group (NL group) and the ligation group (L group) but a trend of difference was showed between this two group and the control group (Figure 4C). Four weeks after the periodontal treatment, heatmaps clearly identified the differences in gut microbial community clustering between non-surgical periodontal treatment group (NL group) and ligation group (L group) (Figure 4D). Significantly different species at all level among the three groups at 5 and 8 weeks were summarized in heatmaps (Figures 4E,F). The samples of the control group were distinctly grouped into one cluster while the NL group and L group were grouped into another cluster 1 week after the non-surgical periodontal treatment (Figure 4E). Subsequently, 4 weeks after the periodontal treatment, the sample of the NL group and the Con group were clustered together while L group were distinctly clustered into another group (Figure 4F), which means NL group became more similar to the control group compared to the L group over time.

### Non-surgical Periodontal Treatment Improve the Intestinal Mucosal Barrier Impaired by Periodontitis in apoE^−/−^ Mice

**Figure 5:**
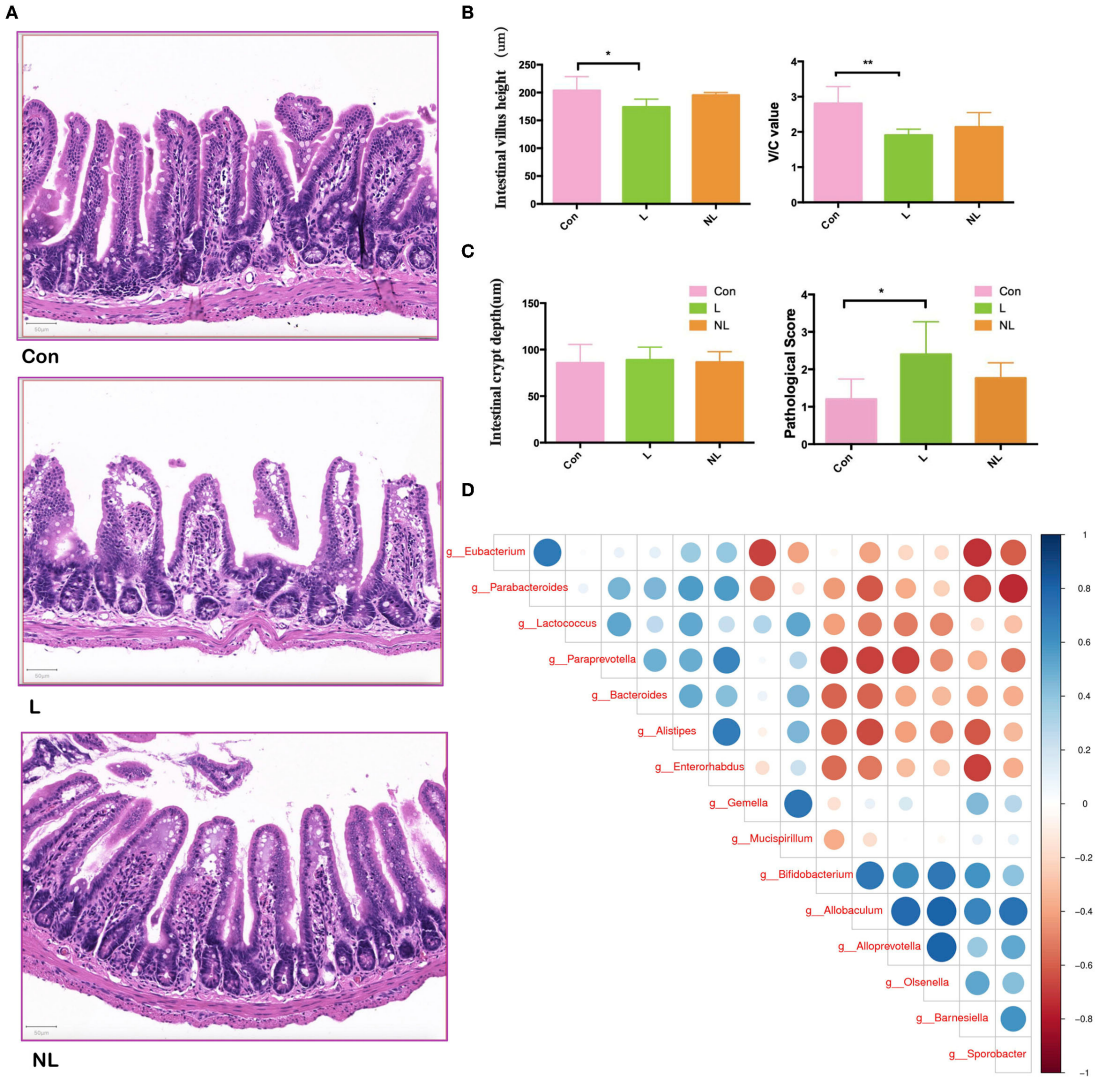
Non-surgical periodontal treatment improve the Intestinal Mucosal Barrier Function of apoE^−/−^ mice with periodontitis. **(A)** Representative images of hematoxylin and eosin staining indicating the intestinal barrier integrity of the ileum (scale bar = 50 μm) and **(B)** quantitative analysis of the morphologic features of the intestinal villus of the ileum. **(C)** The mucosal injury scores in the groups. **(D)** Spearman correlation analysis of the three groups. Bar graphs: Values are presented as mean ± SD. *n* = 5–8 per group. *P < 0.05, **P < 0.01. V/C, intestinal villus height to intestinal crypt depth; Con, control group; L, Ligation group; NL, non-surgical periodontal treatment group.

The intestinal barrier at 8 weeks was analyzed by morphologic evaluation of intestinal villi and mucosal injury scores. Ligature-induced-periodontitis mice showed a significantly compromised intestinal barrier, as reflected by decreased intestinal villus height and V/C (Figures 5A,B; *p* < 0.05). Intestinal crypt depth also increased after periodontitis though the difference was not significant (*p* > 0.05). As shown in Figure 5C, compared with the control group, the mucosal injury scores were significantly increased in the L group (*P* < 0.05), indicating intestinal mucosal injury after periodontitis. Non-surgical periodontal treatment showed a tendency to restore intestinal barrier, as reflected by increased intestinal villus height and V/C, as well as decreased intestinal crypt depth and mucosal injury scores compared to the L group though these differences were not significant (*p* > 0.05).

Besides, LEfSe analysis was used to determine the key alteration of gut flora in mice. (*P* < 0.05, LDA score > 2.0). The results emphasized the role of a butyrate-producing bacteria *Eubacterium*. This specific genus was found abundant in the control group. Eight weeks after ligation, it was removed from gut microbiota. Four weeks after non-surgical periodontal treatment, it was again found to colonize and prosper in gut (Table 1). Furthermore, The Spearman correlation heat map analyzed the important patterns and relationships among the dominant taxa. There were positive correlations between *Eubacterium* and the genus enriched in the control group and negative correlations between *Eubacterium* and the genus enriched in the L group (Figure 5D).

**Table 1:** Significantly altered microbiota of *Eubacterium* at 8 weeks in three groups.

**Taxonname**	**Mean (F-8w-C)**	**Mean (F-8w-L)**	**Mean (F-8w-NL)**	***p*-value**	**fdr**
g__Eubacterium	0.000426617	0	0.000386429	0.003002526	0.096297222

*F-8w-C, control group; F-8w-NL, non-surgical periodontal treatment group; F-8w-L, ligation group; g, genus*.

### Alteration in the Taxa of Gut Microbiota After Ligature-Induced-Periodontitis and Non-surgical Periodontal Treatment

**Figure 6:**
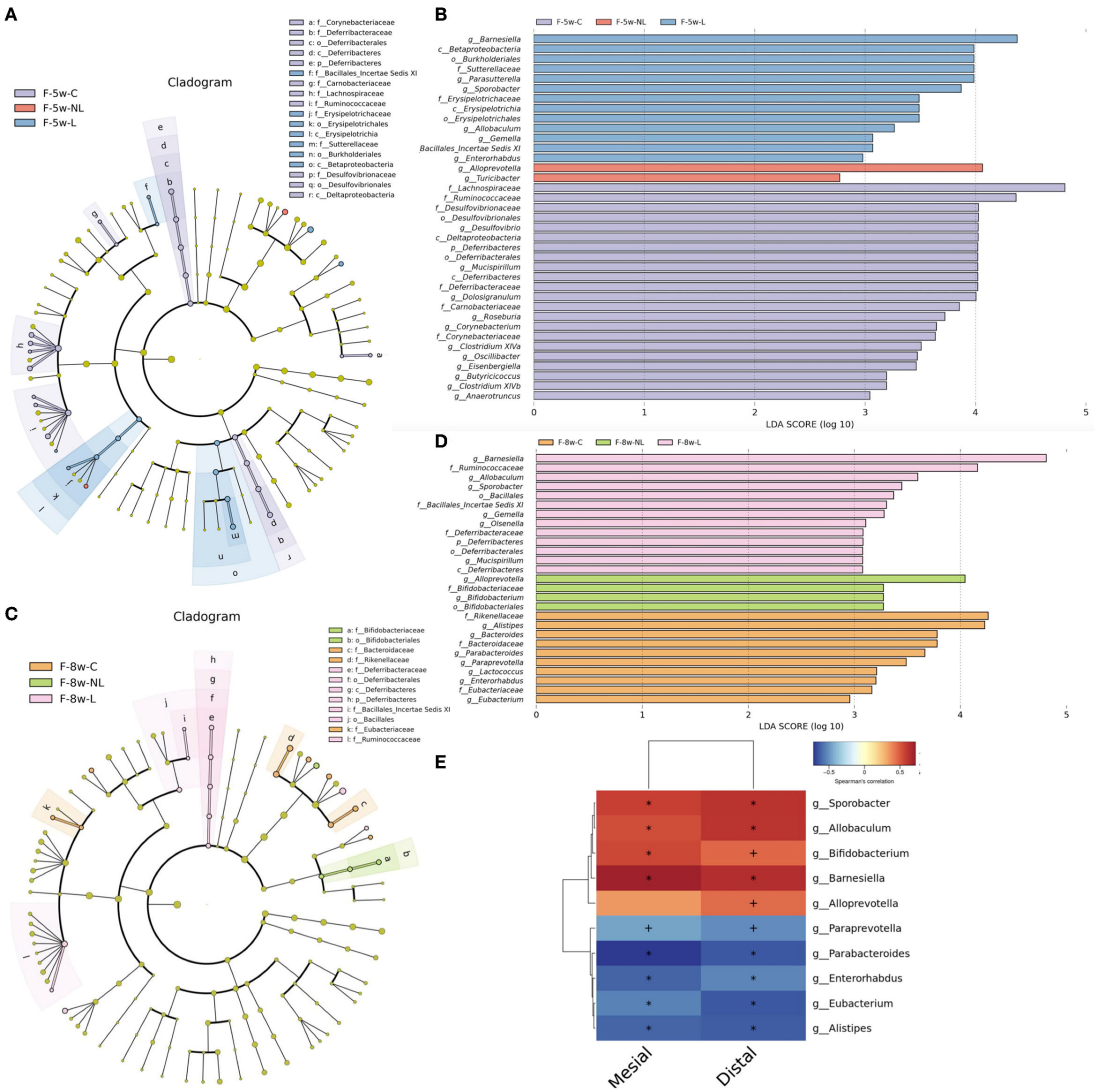
Taxonomic differences and correlation analysis of gut microbiota in Con group, L group, and NL group. Cladogram using LEfSe method indicating the phylogenetic distribution of gut microbiota in three groups at 5 weeks **(A)** and 8 weeks **(C)**. LEfSe analysis revealed significant bacterial differences in gut microbiota among the three groups at 5 weeks **(B)** and 8 weeks **(D)**. The LDA scores (log10)>2 and *P* < 0.05 are listed. **(E)** Heatmap of Spearman correlation analysis between the altered genera and alveolar bone resorption. Distal, distal bone loss; Mesial, mesial bone loss; LDA, Linear discriminant analysis; o, order; f, family; g, genus; F-5w-C, control group; F-5w-NL, non-surgical periodontal treatment group; F-5w-L, ligation group; F-8w-C, control group; F-8w-NL, non-surgical periodontal treatment group; F-8w-L, ligation group.

In order to identify which species are responsible for the significant differences, LEfSe algorithm and Kruskal–Wallis test were used. We used a logarithmic LDA score cutoff of 2.0 to identify important taxonomic differences among three groups. The results suggested that *Gemell, Allobaculum, Barnesiella, Sporobacter* was significantly enriched in the Ligation group (L group) both 5 and 8 weeks after ligation whereas decreased in the NL group after non-surgical periodontal treatment. While the relative abundances of genera *Turicibacter* and *Bifidobacterium* displayed the most increase 1 and 4 weeks after non-surgical periodontal treatment, respectively (Figures 6A–D). In order to analyze the relationship between the gut microbiota and alveolar bone resorption, we performed a correlation analysis between the abundance of significantly altered bacteria at the genus level and alveolar bone resorption at 8 weeks. The results showed that significantly enriched genus in the L group such as *Allobaculum, Sporobacter*, and *Barnesiella* was positively related to the mesial and (or) distal bone loss. The significantly enriched genus *Paraprevotella, Eubacterium, Parabacteroides, Alistipes*, and *Enterorhabdus* in the control group was negatively correlated with the mesial and distal bone loss (Figure 6E).

### Ligature-Induced-Periodontitis and Non-surgical Periodontal Treatment Altered Gut Microbial Function of apoE^−/−^ Mice

**Figure 7:**
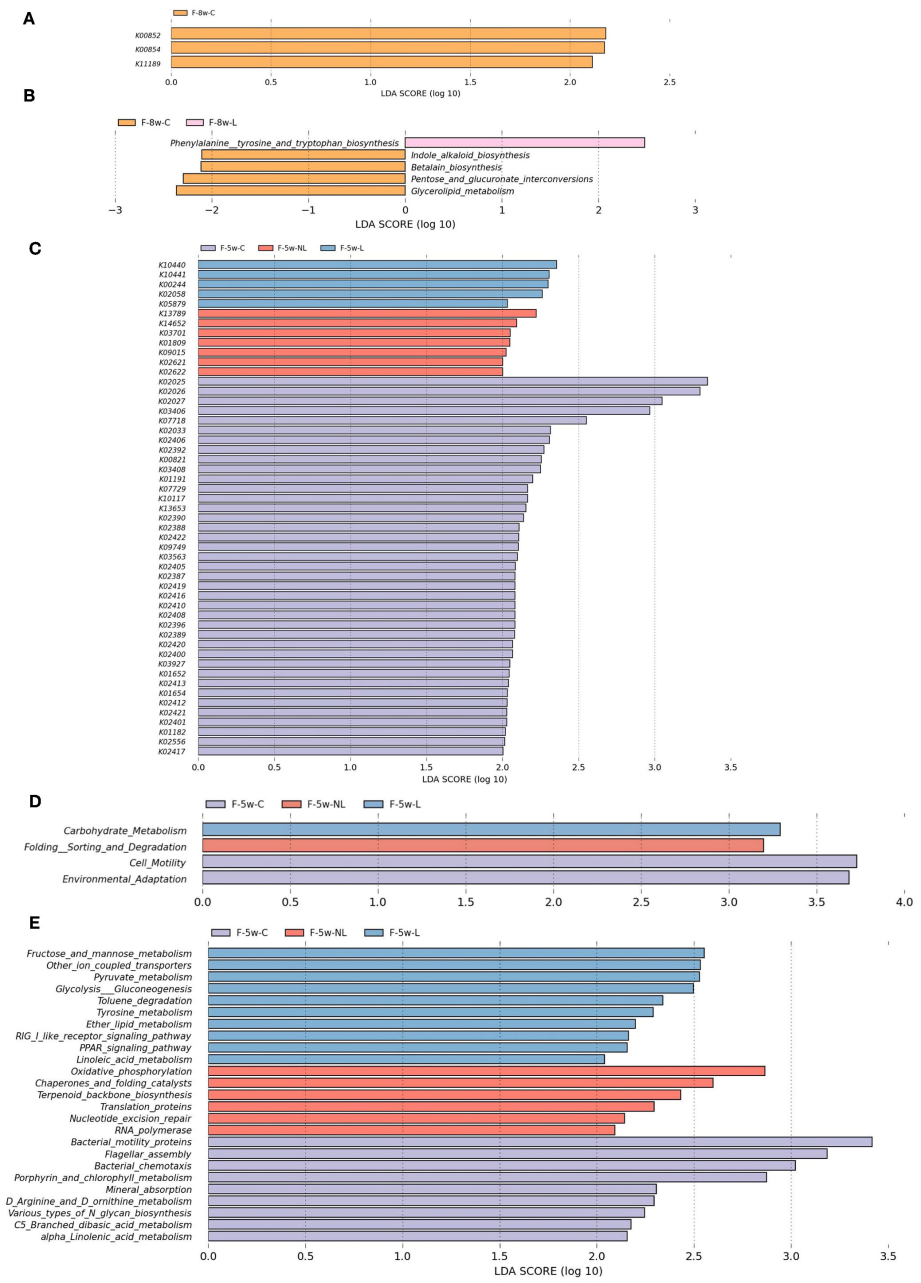
Functional predictions for the intestinal microbiome of the ligation group, the non-surgical periodontal treatment group and the control group at 5weeks and 8weeks. The KOs with significantly different abundances at 5 weeks **(C)** and 8 weeks **(A)** are shown. **(D)** Significant KEGG pathways at level 2 at 5 weeks. Significant KEGG pathways at level 3 at 5 weeks **(E)** and 8 weeks **(B)**. The LDA scores (log10)>2 and *P* < 0.05 are listed. F-5w-C, control group; F-5w-NL, non-surgical periodontal treatment group; F-5w-L, ligation group; F-8w-C, control group; F-8w-NL, non-surgical periodontal treatment group; F-8w-L, ligation group; KO, KEGG orthologs.

PICRUSt based on closed-reference OTU was used to predict the abundances of functional categories according to the the Kyoto Encyclopedia of Genes and Genomes (KEGG) ortholog (KO) database. A LEfSe analysis was performed to investigate whether the differences in gut microbiome composition also had functional consequences concerning the expression of certain genes. A total of 51 KOs were identified with significantly different abundances in the gut microbiome among three groups 1 weeks after non-surgical periodontal treatment (Figure 7C) and 3 KOs were identified 4 weeks after periodontal treatment (Figure 7A). At level 2 of KEGG pathways, Carbohydrate Metabolism were elevated in the L group (F-5w-L) at 5 weeks. Conversely, Folding Sorting and Degradation was predicted at higher levels in the microbiota from the non-surgical periodontal treatment group (F-5w-NL) (Figure 7D). However, no distinct pathway was found at 8 weeks at level 2. In the level 3 KEGG pathways, at 5 weeks, Fructose and mannose metabolism, Other ion coupled transporters, Pyruvate metabolism, Glycolysis Gluconeogenesis, Toluene degradation, Tyrosine metabolism, Ether lipid metabolism, RIG I like receptor signaling pathway, PPAR signaling pathway, Linoleic acid metabolism were higher in the gut microbiome of the L group (F-5w-L), whereas the microbial gene functions related to Oxidative phosphorylation, Chaperones, and folding catalysts, Terpenoid backbone biosynthesis, Translation proteins, Nucleotide excision repair, RNA polymerase were higher in the intestinal microbiome of the non-surgical periodontal treatment group (F-5w-NL) (Figure 7E). At 8 weeks, Phenylalanine tyrosine and tryptophan biosynthesis was higher in the gut microbiome of the L group (F-8w-L) (Figure 7B).

## Discussion

In this study, we showed that periodontal disease induced disturbance of gut microbiota in apoE^−/−^ mice with hyperlipidemia. Microbiological shift implicates functional alteration and therefore altered inflammation status in intestines. Non-surgical periodontal treatment triggered modulation of gut microbiota, helping recovery to a healthy microbiome situation. Meanwhile, non-surgical periodontal treatment strengthened the intestinal mucosal barrier which was impaired by periodontitis, resulting in a stronger non-specific immune function.

In our study, apoE^−/−^ mice with periodontal disease after 4 weeks of ligation established a disturbed gut microbiome. Lower alpha-diversity in the gut microbiome was observed in the experimental periodontitis group compared with control group, although no significant difference between groups was found. Other investigation also showed that patients with periodontal diseases tend to present reduced diversity in the gut microbiota (Lourenςo et al., 2018). In accordance with these data, some findings have shown that lower alpha diversity is a reliable indicator of disease-associated dysbiosis (Turnbaugh et al., 2009; Cho and Blaser, 2012; Langille et al., 2013; Duvallet et al., 2017). Besides, we found that Firmicutes increased in abundance in the periodontitis group compared to the control group. Lourenςo et al. shows a tendency of Bacteroidetes to decrease and Firmicutes to increase in individuals with chronic periodontitis compared to the individuals presenting periodontal health (Lourenςo et al., 2018). Higher Firmicutes/Bacteroidetes ratio in the gut microbiome with other systemic inflammatory conditions has also been reported (Ley et al., 2005; Koren et al., 2010; Nakajima et al., 2015) corroborating our results.

According to Lourenςo et al. it was not possible to distinguish participants to chronic periodontitis group and healthy control group based on the β-diversity of the gut microbiota (Lourenςo et al., 2018). Yet in our study, significant differences were found in β-diversity between experimental periodontitis group and control group based on the unweighted UniFrac distance. This might partially due to the high level of “between-individual” differences in microbial community (Costello et al., 2009). Indeed in human the diversity of intestinal microflora could be affected by multiple factors including diet, smoking, obesity, etc (Lourenςo et al., 2018). We used mice in our experiment and strictly controlled the diet, living environment of the mice. All of the mice live in the same environment and eat the same food. Thus, confounding factors as diet, smoking, obesity, and stress were ruled out. Additionally, it is well-known that apoE-deficient mice have hypercholesterolemia (Zhang et al., 1992). The gut microbiota diversity and composition were remarkably changed in apoE KO mice compared with WT mice on normal chow (Liu et al., 2018). Thus, periodontitis might be more likely to cause intestinal bacterial disorder under hyperlipidemia, which means that the gut microbiota might be more susceptible to interference if disturbed.

Intestinal villus height, crypt depth, V/C, and mucosal injury scores are used to evaluate the integrity of intestinal morphology function (Wen et al., 2019). Detrimental agents, for instance, bacterial toxins, and metabolites can damage the gut epithelial wall once dysbiosis of the microbiota occurs and noxious bacteria become predominant (Cho and Blaser, 2012). Corroborating our results that the intestinal barrier in L group impaired while the gut microbiota were disturbed. We also found that butyrate-producing bacteria *Eubacterium* (Louis et al., 2010; Moens et al., 2017) disappeared in the L group. Fructans-containing diet which fermentation products are mainly short-chain fatty acids increased jejunal villus height and crypt depth in rats (Kleessen et al., 2003). Koch et al. Reported that butyrate supplementation elevated growth of the small intestinal mucosa (Koch et al., 2019). Thus, *Eubacterium* may also play a pivotal role in the protction of intestinal barrier in our study. Besides, butyric acid has a positive influence on the mucosal barrier integrity and anti-inflammatory effect by decreasing proinflammatory cytokine concentrations, furthermore, it stimulates the maturation and proper differentiation of colonocytes. Bacteria that produce butyric acid suppress growth of neoplastic cells and cause their apoptosis (Rakowska et al., 2016). Additionally, it provides energy to the gut epithelium and regulates host-cell responses (Louis et al., 2010). Therefore, *Eubacterium* might play a key role in relating periodontal diseases to systemic diseases.

Commensal bacteria in gut has significant functions. For example, it helps strengthen intestinal integrity, occupy the niche, and stimulating the immune system which protects from the proliferation of potential pathogens (Rakowska et al., 2016). Yet *Gemell, Allobaculum, Barnesiella, Sporobacter* were significantly enriched after ligation in our study. Hence, they may also have an important impact on the relation of periodontal diseases and systemic diseases. Lourenςo et al. characterize the gut microbiome of individuals with different periodontal conditions and found that a subset of OTUs significant correlate with bleeding and periodontal tissue destruction (Lourenςo et al., 2018). In accordance with these data, we observed that most genus enriched in the L group and the control group correlated with the mesial and distal bone loss. 16S rRNA gene sequencing only categorized microbiota at genus level so that more detailed information at species level cannot be discovered. In addition, the abundance alteration of some microbes could not be properly explained due to the fact that microbiota is characterized by transient stability and dynamic balance. Shotgun metagenome analysis can provide deeper analysis of species. Thus, shotgun metagenomics would be needed to study microbiota alteration in more detail.

In the current study, non-surgical periodontal treatment modulates the gut microbiota. The composition of intestinal microbiome was significantly different after non-surgical periodontal treatment. Interestingly, 1 week after the periodontal treatment, there was no apparent distinction between the non-surgical periodontal treatmen (NL group) and the ligation group (L group) while the control group could clearly distinguished from the NL group and the L group in the heatmap which evaluate the similarity of each sample composition. Contrarily, 4 weeks after the periodontal treatment, notable differences could see between the non-surgical periodontal treatment group (NL group) and the ligation group (L group). The results indicated that the intestinal microbiota gradually change over time after the non-surgical periodontal treatment. Furthermore, the heatmaps which summarized significantly different species at all level shows that the non-surgical periodontal treatment group (NL group) and the control (Con group) were clustered together while L group were distinctly clustered into another group 4 weeks after the periodontal treatment. These findings suggested that the intestinal micobiota restored partially toward the healthy state before periodontitis. Besides, Non-surgical periodontal treatment showed a tendency to improve intestinal barrier. Butyrate-producing bacteria *Eubacterium* was also increased after the non-surgical periodontal treatment in our study. So the periodontal treatment is very important for periodontitis patients especially with systematic diseases associated with intestinal microbiota.

Previous studies reported altered serum lipid levels in patients with periodontitis and hyperlipidemia after periodontal treatment (Duan et al., 2009; Fu et al., 2016). However, in our study, serum lipid levels had no significant difference among three groups. It might because that the experiment time–one month after periodontal treatment was not long enough to affect the serum lipid levels. Or it may because that the gut microbiota disturbed by periodontitis had not fully recovered. It is apparent that larger longitudinal clinical trials are needed to determine the impact of longer periodontal treatment on the improvement of systemic health.

Our results also showed that periodontitis and periodontal treatment display different functional profiles of the gut microbiome. Shotgun metagenome analysis can provide more detailed information in the functional analysis. Therefore, shotgun metagenomics would be needed to study the functional changes in the intestinal microbiota after periodontitis and periodontal treatment in more detail.

In conclusion, our study confirmed that gut microbiota dysbiosis occurs in apoE^−/−^ mice with periodontitis and was partially resolved after non-surgical periodontal treatment. Non-surgical periodontal treatment modulates the intestinal mucosal barrier which was impaired by periodontitis. Periodontitis and non-surgical periodontal treatment altered gut microbial function of apoE^−/−^ mice. We also found correlations between alveolar bone resorption and specific microorganisms of the gut microbiota. This study also provides the basis for further mechanistic and interventional studies to prevent or reverse the systemic diseases associated with gut microbiota and periodontitis. However, there is a limitation of this study due to the sequencing method we have chosen. 16S rRNA gene sequencing detected gut microbiota community change at genus level. To achieve more accurate assignment, shotgun metagenomics are needed in further study. Besides, Large clinical studies using metagenomics sequencing are also needed in the future.

## Data Availability Statement

Data can be found in NCBI (SRA accession: PRJNA603416) - https://www.ncbi.nlm.nih.gov/sra/?term=PRJNA603416.

## Ethics Statement

The animal study was reviewed and approved by the Animal Ethics Committee of Nanjing University.

## Author Contributions

YH and FY designed the study. YH did the experiments with the assistance of LL. YH was responsible for the data analyses and wrote the manuscript. BL, LL, and YL assisted in data analyzing. YL and YZ contributed to the revisions. All authors read and approved the final manuscript.

## Conflict of Interest

The authors declare that the research was conducted in the absence of any commercial or financial relationships that could be construed as a potential conflict of interest.
